# Valproate Administered after Traumatic Brain Injury Provides Neuroprotection and Improves Cognitive Function in Rats

**DOI:** 10.1371/journal.pone.0011383

**Published:** 2010-06-30

**Authors:** Pramod K. Dash, Sara A. Orsi, Min Zhang, Raymond J. Grill, Shibani Pati, Jing Zhao, Anthony N. Moore

**Affiliations:** 1 Departments of Neurobiology and Anatomy, and Neurosurgery, The University of Texas Health Science Center at Houston, Houston, Texas, United States of America; 2 Department of Integrated Biology and Pharmacology, The University of Texas Health Science Center at Houston, Houston, Texas, United States of America; 3 Center for Translation Injury Research, The University of Texas Health Science Center at Houston, Houston, Texas, United States of America; University of North Dakota, United States of America

## Abstract

**Background:**

Traumatic brain injury (TBI) initiates a complex series of neurochemical and signaling changes that lead to pathological events including neuronal hyperactivity, excessive glutamate release, inflammation, increased blood-brain barrier (BBB) permeability and cerebral edema, altered gene expression, and neuronal dysfunction. It is believed that a drug combination, or a single drug acting on multiple targets, may be an effective strategy to treat TBI. Valproate, a widely used antiepileptic drug, has a number of targets including GABA transaminase, voltage-gated sodium channels, glycogen synthase kinase (GSK)-3, and histone deacetylases (HDACs), and therefore may attenuate a number of TBI-associated pathologies.

**Methodology/Principal Findings:**

Using a rodent model of TBI, we tested if post-injury administration of valproate can decrease BBB permeability, reduce neural damage and improve cognitive outcome. Dose-response studies revealed that systemic administration of 400 mg/kg (i.p.), but not 15, 30, 60 or 100 mg/kg, increases histone H3 and H4 acetylation, and reduces GSK-3 activity, in the hippocampus. Thirty min post-injury administration of 400 mg/kg valproate improved BBB integrity as indicated by a reduction in Evans Blue dye extravasation. Consistent with its dose response to inhibit GSK-3 and HDACs, valproate at 400 mg/kg, but not 100 mg/kg, reduced TBI-associated hippocampal dendritic damage, lessened cortical contusion volume, and improved motor function and spatial memory. These behavioral improvements were not observed when SAHA (suberoylanilide hydroxamic acid), a selective HDAC inhibitor, was administered.

**Conclusion/Significance:**

Our findings indicate that valproate given soon after TBI can be neuroprotective. As clinically proven interventions that can be used to minimize the damage following TBI are not currently available, the findings from this report support the further testing of valproate as an acute therapeutic strategy.

## Introduction

The cognitive and behavioral dysfunctions associated with traumatic brain injury (TBI) are thought to be due to both the initial injury, and a series of progressive damages and secondary pathologies [1]. These include altered homeostasis due to disruption of the blood-brain barrier (BBB), excessive release of excitatory neurotransmitters, axonal and dendritic disruptions, neuroinflammation, post-traumatic seizures (PTS), and cell death [2]–[5]. PTS are prominent secondary pathologies [6] that can cause excessive release of excitatory neurotransmitters, alterations in blood pressure, decreased oxygen delivery, elevations in intracranial pressure, and an increase in neuronal death following TBI. Based on the complexity of the responses of the brain to trauma, a recent National Institutes of Health working group on TBI recommended either combination therapies or the evaluation of agents that act on multiple mechanisms be evaluated as treatment options for TBI [7].

Valproate [2-propylpentanoic acid] (VPA) is one of the most commonly prescribed antiepileptic medications that has been shown to reduce the neuronal damage associated with epileptic activity. For example, in a model of rodent *status epilepticus*, VPA markedly decreased neuronal damage in the hippocampal formation and improved neurological and memory functions [8]. Following TBI, VPA has been shown to be effective in treating early-, but not late-onset PTS [9]. It is thought that the antiepileptic activity of VPA results from a combination of its influence on a number of targets in the central nervous system including inhibition of GABA transamination, reduction of NMDA-mediated neuronal excitation, inhibition of histone deacetylases (HDACs) and glycogen synthase kinase (GSK)-3, and blockade of voltage-gated sodium and T-type calcium channels [10]. As altered GABAergic and glutamatergic neurotransmission, decreased histone acetylation, and calcium entry have all been implicated in TBI pathology, we questioned if VPA can be used as a treatment option following TBI.

In this report, we demonstrate that post-injury administration of VPA causes a dose-dependent improvement in behavioral function following experimental TBI. This improvement was associated with a decrease in cortical contusion volume, improved BBB integrity, and preservation of hippocampal dendritic integrity. Our findings support the use of VPA as a treatment for TBI, and suggest its use merits further clinical investigation.

## Materials and Methods

### Materials

Male Sprague-Dawley rats (275–300 g) were purchased from Charles River Laboratories (Wilmington, MA). Sodium valproate was purchased from Sigma Aldrich (St. Louis, MO). Antibodies to acetylated histone H3, acetylated histone H4, and total histone H4 were obtained from Millipore (Billerica, MA). Phospho ß-catenin (Ser^33/37^), total ß-catenin, phospho-ERK1/2 (Thr^202^/Tyr^204^, Thr^185^/Tyr^187^), total ERK1/2 and double-cortin antibodies were purchased from Cell Signaling Technology (Danvers, MA). Anti-MAP2 antibodies were obtained from Covance (Princeton, NJ) and the CD68 antibody purchased from Serotec (Raleigh, NC).

### Production of brain injury

All experimental procedures were approved by the Animal Welfare Committee of the University of Texas Health Science Center at Houston and were conducted in accordance with the recommendations provided in the *Guide for the Care and Use of Laboratory Animals*. Protocols were designed to minimize pain and discomfort during the injury procedure and recovery period. An electromagnet-driven controlled cortical impact (CCI) device was used to cause brain injury as previously described [11]–[13]. Briefly, animals were anesthetized using 5% isofluorane with a 1∶1 O_2_/N_2_O mixture, mounted on a stereotaxic frame, and a midline incision made. For BBB, a unilateral 6-mm crainiectomy was prepared midway between the bregma and lambda with the medial edge of the craniectomy 1-mm lateral to the midline as described by Meaney et al [14]. For behavior, bilateral craniectomies were produced. Rats received a single impact (2.7 mm deformation) on the right parietal lobe with an impact velocity of 6 meters/sec. Core body temperature was maintained at 37–38°C by use of a heating pad. The animals were given time to recuperate in a warming chamber before being returned to their home cages. Animals were weighed daily after the injury for the first 3 days, then weekly thereafter.

### Drug preparation and administration

Sodium valproate (VPA) was dissolved in sterile saline to a final concentration of 60 mg/ml (for the 400 mg/kg dose) or 15 mg/ml (for the ≤100 mg/kg doses). For testing the influence of VPA on cognitive function, animals were injured then injected (i.p.) with the designated amount of VPA or an equal volume of vehicle 30 minutes or 3 hours post-injury, and every 24 hours thereafter for the first 5 days post-injury. For biochemical analysis, uninjured rats were given a single i.p. injection of VPA (15, 30, 60, 100 or 400 mg/kg) or vehicle. For experiments involving suberoylanilide hydroxamic acid (SAHA), the drug was prepared as a 100 mg/ml stock in DMSO. Final DMSO concentration for injection was 40% in saline.

### Sample Preparation

Hippocampal tissues were quickly removed while submerged in ice cold artificial cerebral spinal fluid (10 mM HEPES pH 7.2, 1.3 mM NaH_2_PO_4_, 3 mM KCl, 124 mM NaCl, 10 mM dextrose, 26 mM NaHCO_3_ and 2 mM MgCl_2_) containing phosphatase inhibitors (2 mM sodium fluoride, 2 mM sodium molybdate and 1 mM sodium orthovanadate). Tissues were homogenized (20 strokes) in 10 volumes of a buffer containing 10 mM Tris pH 7.4, 1 mM EGTA, 1 mM EDTA, 0.5 mM DTT, phosphatase inhibitors (0.1 µM okadaic acid and 1 mM sodium orthovanadate) and protease inhibitors (1 mM PMSF and 10 µg/ml leupeptin) using a motorized teflon-glass homogenizer. The samples were immediately aliquoted and frozen at −80°C.

### Western Blotting

Hippocampal tissue extracts were sonicated (5 pulses of 1 second each) using a Sonics Vibracell sonicator (Sonics & Materials, Inc., Newtown, CT) and a 0.4 mm diameter probe. The amount of protein in each sample was determined by a Bradford assay using bovine serum albumin (BSA) as the standard. Samples were denatured at 95°C for three minutes in 1× NuPage SDS sample buffer (Invitrogen, Carlsbad, Calif.). Equal amounts of protein were loaded, electrophoresed, and transferred to Immobilon-P membranes (Millipore, Billerica, MA) using the NOVEX X-Cell II system (Invitrogen, Burlingame, CA) and the buffers provided by the vendor. Membranes were blocked overnight in 5% BSA, followed by a three-hour incubation in primary antibodies (0.5 µg/ml) at room temperature. Membranes were then washed and incubated at room temperature with alkaline phosphatase (AP)-conjugated secondary antibodies for one hour as recommended by the vendor (Vector Laboratories, Burlingame, CA). Immunoreactivity was detected using a chemiluminescence system and quantified using *Image J* (freely available through NIH).

### Measurement of BBB permeability

BBB permeability was assessed by measuring the extravasation of Evans blue (EB) dye as described previously [15], [16]. 3% EB in saline was injected slowly through the Jugular vein (4 ml/kg) and allowed to circulate for 1.5 hr. Following the circulation time, animals were transcardially perfused with 1X PBS followed by 4% paraformaldehyde. The brains were removed, cerebral hemispheres were separated and 2 mm-thick sections were prepared for each hemisphere. The brain sections from each hemisphere were incubated in 5 ml 55°C formamide for 24 hr. At the end of the incubation, tissues were removed and the formamide solution was centrifuged at 20,000×g for 20 min in an Eppendorf centrifuge. The supernatant solution was collected and the optical density at 620 nm was measured to determine the relative amount of EB dye in each sample.

### Assessment of motor function

All behavioral tests were conducted by an experimenter blind to the treatment groups. Two different motor skill tasks (beam balance and paw placement) were used to determine animals' motor performance following injury [17], [18] on days 1–3 post-injury. For beam balance, rats were pre-assessed by placing them on a narrow wooden beam (1.5 cm wide) and measuring the duration they remained on the beam for up to 60 seconds. Animals were given repeated pre-injury training until capable of balancing on the beam for the entire 60 sec period in 3 consecutive trials. Following injury, animals were given three daily trials during which the length of time spent on the beam was recorded. Paw placement was evaluated by placing the animal on a wire grid (opening size of 2×2 cm) and counting the number of foot faults out of a total of 50 steps. A foot fault was defined as when a front paw misses and appears below the plane of the grid. Paw placement was repeated three times to give an average daily score.

### Assessment of cognitive function

Rats were tested for their cognitive performance using the standard hidden platform version of the Morris water maze [12], [19]–[21]. All animals had recovered from the TBI-associated motor dysfunction prior to performing the cognitive testing. Animals were given 4 consecutive training trials per day for 7 days, with an inter-trial interval (iti) of 4 min. If the animal failed to locate the platform within 60 sec on any given trial, it was led there by the experimenter. Twenty-four hours following the last day of training, animals were tested in a probe trial to measure quadrant preference and platform localization. Movement within the maze was monitored using a video camera linked to tracking software (Ethovision, Noldus Information Technology, Leesbury, VA, USA).

### Contusion volume measurement

Following the completion of the behavioral studies, approximately 28 days following the injury, animals were deeply anesthetized with sodium pentobarbital (100 mg/kg) and transcardially perfused with phosphate buffered saline (PBS) followed by 4% paraformaldehyde. Brains were removed, post-fixed overnight in perfusant, then cryoprotected in a 30% sucrose solution. Cortical tissue loss was estimated essentially as described previously [22], by experimenters kept blind with respect to the treatment groups. In brief, cryosections (40 µm thickness) spanning the rostral-caudal extent of the injured cortex were selected and stained with cresyl violet by an experimenter given only the animal's identifier code. Images of the resultant slides were then used for tissue loss measurement by a second experimenter. The area of cortical tissue loss for each section was carefully outlined using *Adobe Illustrator*, with the area of the resultant outlines quantified by *Image J,* from the National Institutes of Health. Contusion volume was calculated using the equation A_1_(0.5X_1_) + A_2_(0.5X_1_+0.5X_2_) + A_n−1_(0.5X_n−1_ +0.5X_n_) + A_n_(0.5X_n_) where A is the area (mm^2^) of the contusion for each slice, and X is the distance (mm) between two sequential slices. Once the contusion volume had been calculated for each animal, the blind code was broken and group differences assessed.

### Immunohistochemistry

To examine the consequences of TBI on histone acetylation, rats were injured or sham-operated as described in the *Production of cortical impact injury* section. For post-TBI histological evaluation, tissue sections generated for the evaluation of cortical contusion volume were used for immunohistochemistry. Free-floating slices were incubated overnight in primary antibody (0.5–1.0 µg/ml) in TBS containing 2% BSA and 2.5% normal goat serum. Immunoreactivity was detected using species-specific secondary antibodies coupled to Alexafluors. Disruptions in microtubule immunoreactivity were quantified for size and luminosity using digital images of MAP-2 immunoreactivity and *Image J*. Luminosity was calculated as the average of three measurements within a 75×75 µm box randomly placed within the damaged area. Luminosity was normalized within each section by comparing to the luminosity values obtained from adjacent, undamaged areas. For quantification of neurogenesis, the number of doublecortin-positive cells embedded within the granule cell layer of the dentate gyrus were counted in three sections spanning the dorsal hippocampus by an independent observer. The length of the dentate gyrus was measured, and the number of cells/mm calculated. As several studies have shown that cortical impact injury gives rise to widespread cellular and biochemical changes in both the injured and contralateral hemispheres [14], [23]–[25], comparisons were made between injured animals and sham-operated controls.

### Statistical Analysis

For evaluation of behavioral data, repeated measures analyses of variance (two-way or one-way as appropriate) and t-tests were utilized to determine statistical differences. A Holm-Sidak method for multiple comparisons post-hoc test was used to determine data points with significant differences. Dose response western data was evaluated using a one-way ANOVA followed by a Dunnet's *post-hoc* test. Data were considered significant at p≤0.05 and presented as Mean ± Standard Error of the Mean (S.E.M.).

## Results

### Systemic valproate administration increases hippocampal histone acetylation

**Figure 1:**
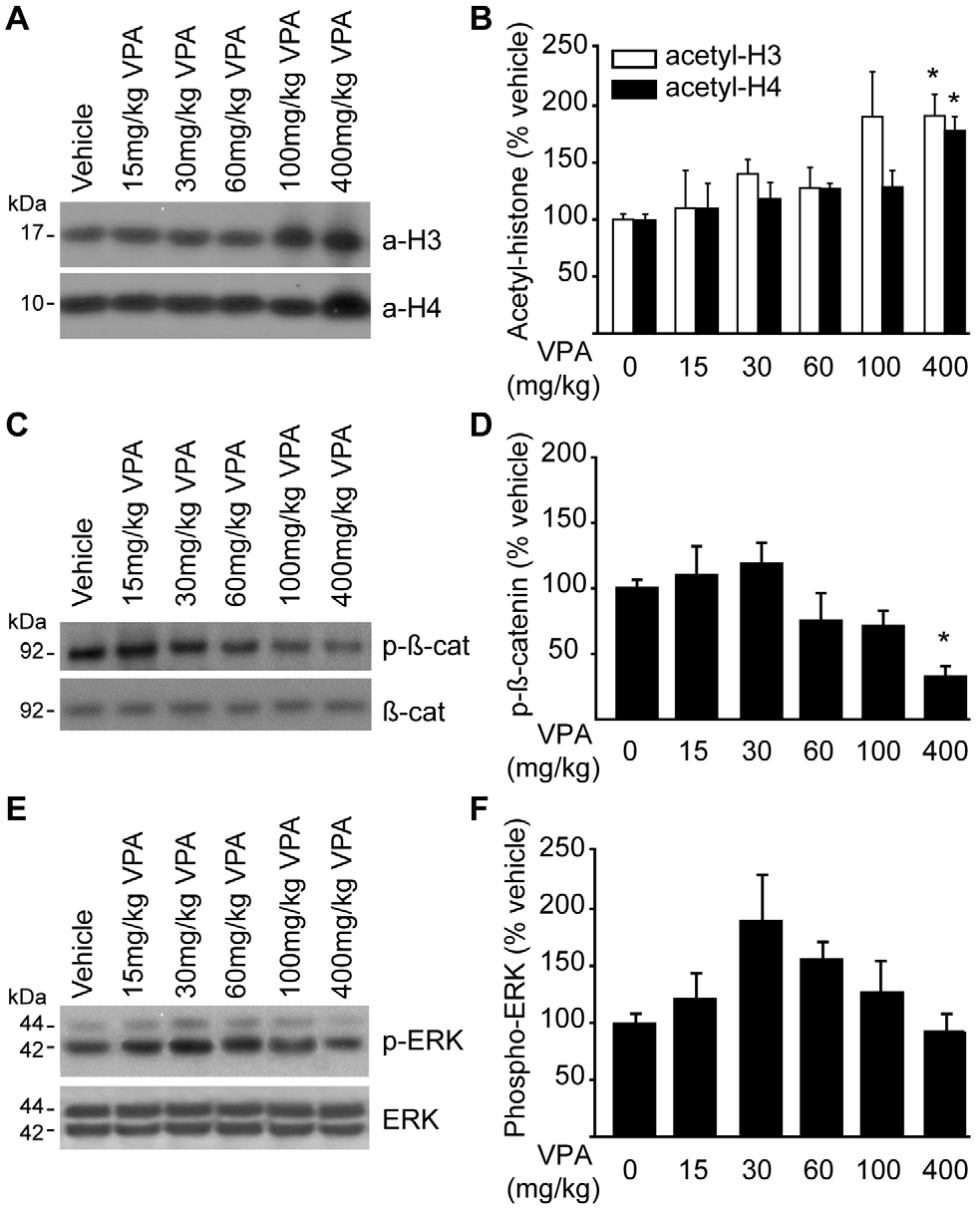
Valproate increases hippocampal histone acetylation, and inhibits GSK-3 activity, in a dose-dependent manner. **A)** Picture of a representative immunoblot showing histone H3 (a-H3) and H4 (a-H4) acetylation following systemic administration of different doses of valproate (VPA). **B)** Summary data for acetylated histone H3 and acetylated histone H4 following different doses of i.p. injected VPA. **C)** Picture of a representative western blot showing phosphorylated ß-catenin (p-ß-cat), and total β-catenin (ß-cat), immunoreactivities following VPA administration. **D)** Summary data showing that VPA decreases β-catenin phosphorylation in a dose dependent manner. **E)** Picture of a representative western blot showing phosphorylated (p-ERK) and total ERK immunoreactivity following VPA administration. **F)** Summary data showing that VPA did not have any significant influence on ERK phosphorylation. Data are presented as mean ± SEM, *,P**≤**0.05.

Valproate (VPA) has been previously demonstrated to be an inhibitor of histone deacetylase (HDAC) [26], [27], and as such preserves the acetylation of histones H2A, H3 and H4 [28]. To examine the dose-response relationship between systemic VPA administration and histone acetylation in the brain, western blots were performed using acetyl-H3 and acetyl-H4 histone antibodies. Rats were i.p. injected with designated amounts of VPA, and then killed 45 min later. Hippocampal protein extracts were prepared for western blots. All western blots were performed using starting material amounts within the linear range of detection. The representative western blots (Figure 1A) and summary data (Figure 1B) show that systemic VPA administration (n = 3/dose) significantly increases both histone H3 and H4 acetylation in the hippocampus (acetyl-H3: one way ANOVA F_(5,15)_ = 2.95, P = 0.047; acetyl-H4: one way ANOVA F_(5,15)_ = 4.89, P = 0.008). These changes occurred in a dose-dependent manner with 400 mg/kg dose significantly increasing hippocampal histone acetylation compared to vehicle-treated controls. No significant changes were detected when the levels of total H3 and H4 were evaluated (H3: one way ANOVA F_(5,15)_ = 1.81, P = 0.172; H4: one way ANOVA F_(5,15)_ = 0.48, P = 0.783). When expressed as a ratio of acetylated:total histone levels, it was confirmed that 400 mg/kg VPA significantly increases the acetylation of both histone H3 (Kruskal-Wallis one-way ANOVA on Ranks P = 0.009) and histone H4 (one way ANOVA F_(5,15)_ = 3.65, P = 0.023).

In addition to its inhibition of HDAC, VPA has been shown to activate the glycogen synthase kinase-3 (GSK-3)/ß-catenin and extracellular signal-regulated kinase (ERK) pathways [10]. ß-catenin is normally phosphorylated by GSK-3 targeting it for ubiquitin-mediated degradation. By inhibiting GSK-3, VPA causes the accumulation of non-phosphorylated ß-catenin that leads to enhanced gene expression via the TCF/LEF family of transcription factors [29]. To examine this consequence of VPA administration, western blots were performed using phospho- and total ß-catenin antibodies. Figure 1C shows a representative western blot of phospho-ß-catenin and ß-catenin immunoreactivity following designated amounts of VPA injections. Figure 1D shows that, similar to histone acetylation, ß-catenin is activated in a dose-dependent fashion following VPA administration (phospho-ß-catenin: one way ANOVA F_(5,15)_ = 5.67, P = 0.004) with a significant decrease observed at the 400 mg/kg dose. When the ratio of phospho:total ß-catenin was used for statistical analysis, the 400 mg/kg dose of VPA was again found to significantly reduce ß-catenin phosphorylation (one way ANOVA F_(5,15)_ = 4.96, p = 0.007). In contrast, no significant change was observed in the phosphorylation (one way ANOVA F_(5,15)_ = 2.04, p = 0.131) or total levels (one way ANOVA F_(5,15)_ = 0.48, p = 0.783) of ERK as a result of VPA treatment at the time point examined (Figures 1E and 1F). No significant difference was detected when phospho-ERK immunoreactivity was normalized with respect to total ERK immunoreactivity (one way ANOVA F_(5,15)_ = 1.49, p = 0.252). Qualitatively similar results were obtained when the ERK1 (p44) and ERK2 (p42) were independently quantified (data not shown).

### Valproate reduces TBI-associated blood-brain barrier permeability

One of the key pathological changes that occurs acutely following brain injury is disruption of the blood-brain barrier (BBB). Recently, Alam and colleagues have reported that acute treatment of rats following hemorrhagic shock with valproate normalizes serum claudin-3 levels, suggesting improved barrier function [30]. To investigate if post-injury administration of VPA can reduce TBI-associated BBB permeability, rats were anesthetized and given a unilateral crainiectomy followed by cortical impact injury (3.3 mm deformation). Thirty min later, rats were randomly divided into either 400 mg/kg VPA (i.p.) or vehicle (0.9% sodium chloride) groups. Forty-eight hours after the injury, animals were infused with Evans Blue dye and brains removed for evaluation of barrier integrity as described previously [31], [32]. Rats treated with VPA (n = 9) were found to have significantly reduced Evans Blue extravasation in their ipsilateral hemisphere than their vehicle-treated counterparts (n = 9/group) (O.D./gram tissue: vehicle = 0.298±0.028; 400 mg/kg VPA = 0.196±0.039; p = 0.039). Minimal Evans Blue was found in the contralateral hemisphere, which did not change as a result of treatment (O.D./gram tissue: vehicle = 0.020±0.010; 400 mg/kg VPA: 0.016±0.006; p = 0.302).

### Valproate improves motor function following TBI

**Figure 2:**
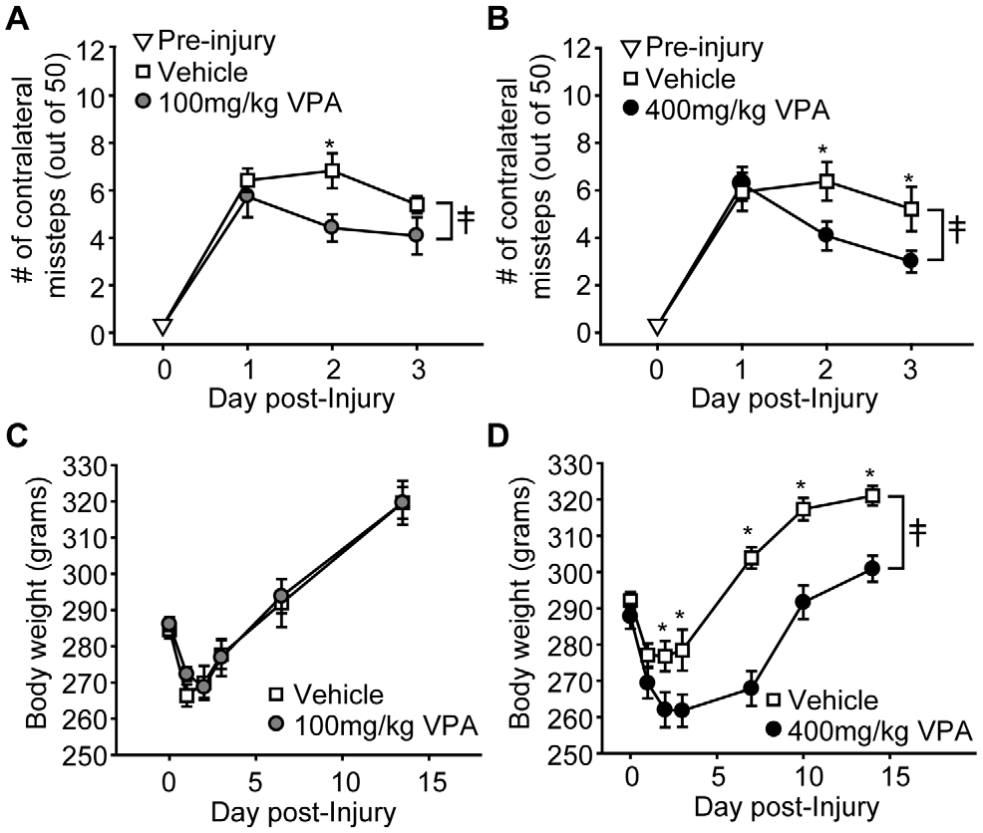
Post-injury valproate administration improves motor skills, but exacerbates injury-associated weight loss. Injured rats treated 30 min post-injury with either **A)** 100 mg/kg valproate (VPA) or **B)** 400 mg/kg VPA had improved motor function as indicated by reduced numbers of contralateral foot faults compared to simultaneously tested vehicle-treated controls. **C)** Post-injury weight loss is not affected by 100 mg/kg VPA. In contrast, **D)** 400 mg/kg exacerbated the degree and duration of weight loss after injury. Data are presented as the mean ± SEM. ≨︀, significant difference by repeated measures two-way ANOVA. *, P≤0.05.

The influence of post-injury VPA injection on vestibulomotor and motor function was tested from days 1–3 post-TBI using the beam balance and foot fault tasks, respectively. For these studies, rats were given bilateral crainiectomies, then injured by a single impact to the right parietal cortex. Thirty min after the injury, rats were i.p. injected with either 100 mg/kg VPA (or vehicle) or 400 mg/kg VPA (or vehicle). Drug administration was continued every 24 hr for the first 5 days of injury. Neither 100 mg/kg VPA (n = 10, repeated measures two-way ANOVA: F_(1,18)_ = 0.08, p = 0.783) nor 400 mg/kg VPA given 30 min post-TBI (n = 8, repeated measures two-way ANOVA: F_(1,15)_ = 2.91, p = 0.109) significantly improved beam balance performance compared to simultaneously tested vehicle controls (n = 10 and n = 9, respectively) (data not shown). Figure 2A shows that the number of contralateral foot faults, on the other hand, was found to be significantly improved as a result of 100 mg/kg VPA treatment (group main effect by repeated measures two-way ANOVA: F_(1,18)_ = 4.38, p = 0.050). This beneficial effect on motor performance was also observed in the 400 mg/kg VPA-treated animals (interaction of group and trial by repeated measures two-way ANOVA: F_(2,28)_ = 3.74, p = 0.036) (Figure 2B). Ipsilateral foot faults were not affected by either treatment dose (data not shown).

One of the common side effects of valproate administration is weight gain [33]. As TBI is associated with acute weight loss, and this weight loss occurs during the time frame of motor skill testing, we questioned if the improved motor skill performance we observed could be due to a reduction in post-trauma weight loss. Figure 2C shows that, contrary to our expectations, post-injury administration of 100 mg/kg VPA had no effect on weight loss (group main effect by repeated measures two-way ANOVA: F_(1,18)_ = 0.02, p = 0.901). At the highest dose tested, 400 mg/kg VPA, a significant worsening of post-injury weight loss was observed (interaction of group and day by repeated measures two-way ANOVA: F_(5,75)_ = 10.40, p<0.001; Figure 2D).

### Intraperitoneal injection of 400 mg/kg, but not 100 mg/kg, valproate improves spatial learning and memory

**Figure 3:**
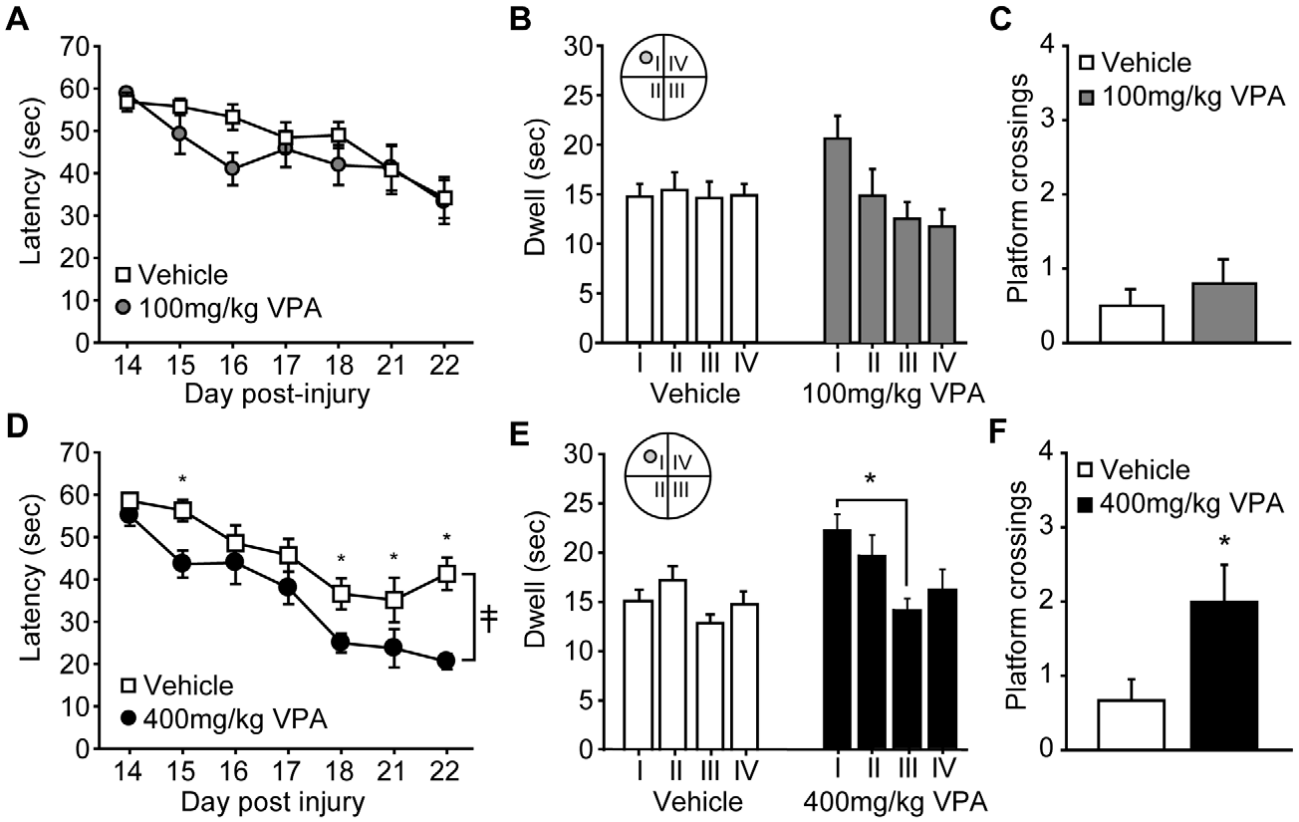
Post-injury administration of 400 mg/kg, but not 100 mg/kg, valproate improves behavioral performance in brain injured rats. Injured rats treated with either valproate (VPA) or vehicle 30 min post-injury were tested in the standard hidden platform version of the Morris water maze. **A)** No difference in water maze performance was detected between the vehicle- and 100 mg/kg VPA-treated injured rats. Similarly, when tested in a probe trial given 24 hr after the completion of training, there was no significant **B)** quadrant preference, nor **C)** difference in number of platform crossings as a result of 100 mg/kg VPA treatment. In contrast, rats treated with 400 mg/kg had **D)** significantly improved water maze performance that was associated with **E)** a preference for the target quadrant (quadrant I) and **F)** significantly more platform crossings during a probe trial given 24 hr after the completion of training. Data are presented as the mean ± SEM. ≨︀, significant group difference by repeated measures two-way ANOVA. *, p≤0.05.

To test the effects of post-injury VPA administration on the cognitive deficits observed following TBI, rats were trained in the hidden platform version of the Morris water maze. Testing began on day 14 post-injury, and consisted of 4 trials/day for a total of 7 days. All animals had recovered from the vestibulomotor and motor deficits by the time water maze performance was tested. Figure 3A shows that injured animals treated with 100 mg/kg VPA did not have learning curves that were significantly different from their vehicle-injected counterparts (repeated measures two-way ANOVA: F_(1,18)_ = 0.92, p = 0.350). When tested in a probe trial 24 hr following training, vehicle-treated, injured animals did not demonstrate a preference for the quadrant in which the platform was located (repeated measures one-way ANOVA: F_(3,27)_ = 0.04, p = 0.989) indicating a lack of memory for the location of the platform (Figure 3B). Although the 100 mg/kg VPA-treated animals displayed improved localization to the target quadrant, their performance did not reach statistical significance (repeated measures one-way ANOVA: F_(3,27)_ = 2.64, p = 0.070). Likewise, Figure 3C shows that the number of platform crossings did not significantly differ between the two groups (Mann-Whitney Rank Sum Test: p = 0.585).

In contrast to that observed for the 100 mg/kg VPA-treated animals, Figure 3D shows that administration of 400 mg/kg VPA significantly improved the performance of injured animals in the water maze (group main effect by repeated measures two-way ANOVA: F_(1,15)_ = 15.03, p = 0.001). Post-hoc analysis revealed that this difference was primarily due to significantly reduced latencies to the hidden platform during the last 3 days of training, suggesting an improved memory for the platform location. Consistent with this suggestion, animals treated with 400 mg/kg VPA performed better in a probe trial given 24 hr following training as indicated by a significant quadrant preference in the VPA-treated animals (repeated measures one-way ANOVA: F_(3,21)_ = 3.11, p = 0.048; Figure 3E) that was not observed in injured rats treated with vehicle (repeated measures one-way ANOVA: F_(3,24)_ = 1.65, p = 0.205). In addition, rats given 400 mg/kg VPA had a significant increase in the number of platform crossings (Mann-Whitney Rank Sum Test: p = 0.046; Figure 3F). Swimming speed was not significantly different between the 400 mg/kg VPA- and vehicle-treated animals (vehicle: 26.27±1.32 cm/sec; 400 mg/kg VPA: 25.0±1.69 cm/sec; P = 0.529).

### Three hour delayed administration of valproate improved motor, but not cognitive, function

**Figure 4:**
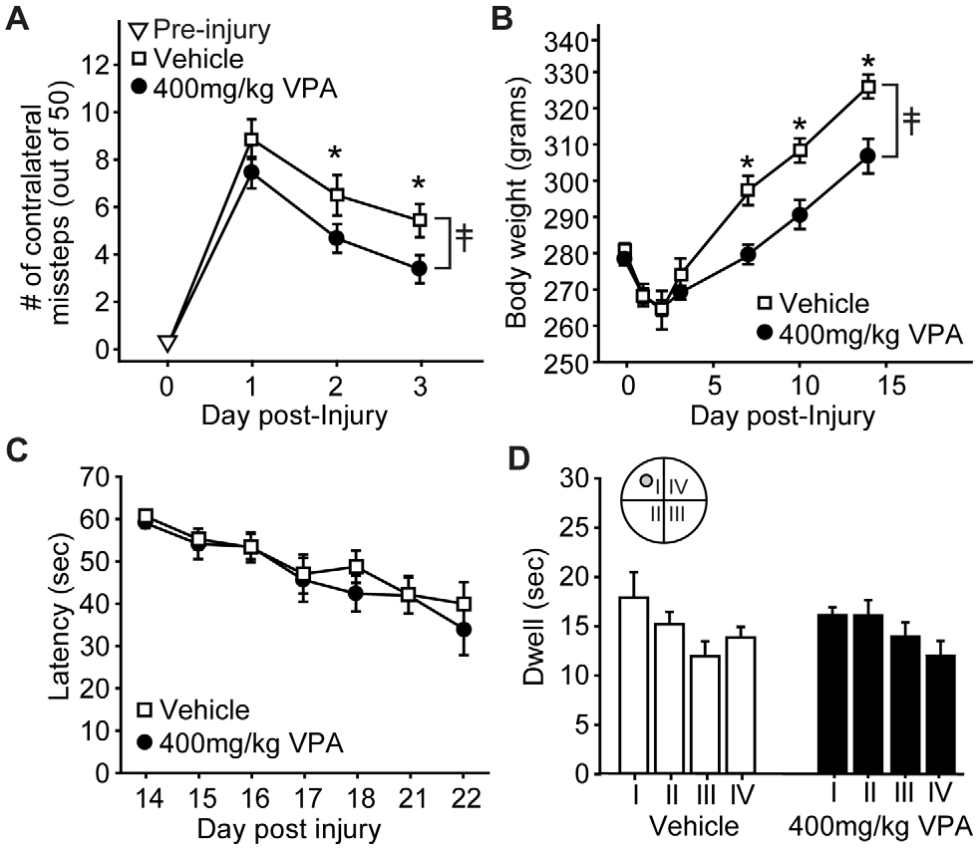
Delayed valproate administration improves motor skills, but does not improve cognitive function. **A)** Injured rats treated 3 hr post-injury with 400 mg/kg valproate (VPA) had improved motor function as indicated by reduced numbers of contralateral foot faults compared to simultaneously tested vehicle-treated controls. **B)** Post-injury weight loss was exacerbated by delayed administration of 400 mg/kg VPA. **C)** No difference in water maze performance was detected between the vehicle- and delayed VPA-treated injured rats. **D)** When tested in a probe trial given 24 hr after the completion of training, there was no significant quadrant preference as a result of delayed VPA treatment. Data are presented as the mean ± SEM. ≨︀, significant group difference by repeated measures two-way ANOVA. *, p≤0.05.

To test if a 3 hr delay can be used for treatment initiation, injured animals were injected with either 400 mg/kg VPA or vehicle then tested in motor and cognitive tasks as described above. Treatment was continued by daily injections for the following 4 days. Figure 4A shows that when the treatment was delayed by 3 hr, improved motor function was still observed, as indicated by significantly fewer contralateral foot faults in the VPA-treated animals (n = 9) than the vehicle-treated controls (n = 11) (repeated measures two-way ANOVA: F_(1,18)_ = 4.47, p = 0.048). Exacerbated weight loss as a result of treatment also persisted in these animals (F_(1,18)_ = 7.15, p = 0.015; Figure 4B). However, no benefit of delayed VPA treatment was observed in water maze learning (repeated measures two-way ANOVA: F_(1,18)_ = 0.37, p = 0.549; Figure 4C), nor in measures of memory such as number of platform crossings (# of crossings: vehicle = 0.73±0.33; 400 mg/kg VPA = 0.78±0.28; p = 0.706) or quadrant preference (repeated measures one-way ANOVA: F_(3,24)_ = 1.63, p = 0.208; Figure 4D) compared to controls.

### HDAC inhibition is not sufficient to mimic the beneficial effects of valproate

**Figure 5:**
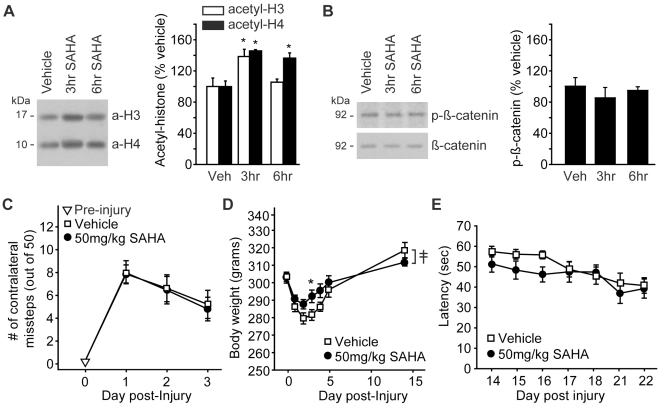
Post-injury administration of SAHA does not mimic the behavioral improvement seen following valproate. **A)** Picture of a representative immunoblot and summary data showing increased histone H3 and H4 acetylation following systemic administration of 50 mg/kg SAHA. **B)** Picture of a representative western blot and summary data showing that the phosphorylation of ß-catenin is not affected by SAHA administration. **C)** Injured rats treated 30 min post-injury with 50 mg/kg SAHA did not have improved motor function, as indicated by numbers of contralateral foot faults, compared to simultaneously tested vehicle-treated controls. **D)** Post-injury weight loss was reduced by 50 mg/kg SAHA. **E)** Injured rats treated with either SAHA or vehicle 30 min post-injury were tested in the standard hidden platform version of the Morris water maze. No difference in water maze performance was detected between the two groups.

Previous reports have indicated that many of the neuroprotective properties of VPA are linked to its HDAC inhibitory activity [34]–[36]. To test if the behavioral improvements observed following 400 mg/kg VPA administration can be mimicked by a more selective HDAC inhibitor, the influence of suberoylanilide hydroxamic acid (SAHA) on TBI-associated motor and cognitive dysfunction was examined. SAHA is a potent inhibitor of class I and class II HDACs that has been used by a number of investigators to assess the role of HDAC activity in pathological conditions [37]–[41]. In order to examine the effectiveness of SAHA on hippocampal histone acetylation, 50 mg/kg SAHA was injected i.p. and protein extracts prepared 3 hr (n = 4) and 6 hr (n = 3) later. This dose of SAHA has been previously demonstrated to significantly increase histone acetylation in the mouse brain [39]. Vehicle-injected rats (n = 4) were used as controls. Figure 5A shows that 50 mg/ml SAHA significantly increased the acetylation of both histone H3 (one-way ANOVA: F_(2,8)_ = 5.61, p = 0.030; acetylated:total ratio: F_(2,8)_ = 4.86, p = 0.042) and histone H4 (one-way ANOVA: F_(2,8)_ = 20.24, p<0.001; acetylated:total ratio: F_(2,8)_ = 7.70, p = 0.014) in the hippocampus. In contrast, no significant influence on ß-catenin phosphorylation was observed at the time points examined (one-way ANOVA: F_(2,8)_ = 0.48, p = 0.635; phosphorylated:total ratio: F_(2,8)_ = 0.17, p = 0.848) (Figure 5B), indicating no effect of SAHA on GSK-3 activity.

To test the behavioral consequences of post-injury SAHA administration, rats were i.p. injected with either 50 mg/kg SAHA (n = 11) or an equal volume of vehicle (n = 11) 30 min after the injury. As was done for VPA, drug administration was continued every 24 hr for the first 5 days of injury. Figure 5C shows that unlike VPA, SAHA did not improve motor function as indicated by similar numbers of contralateral foot faults (repeated measures two-way ANOVA: F_(1,20)_ = 0.03, p = 0.874). No significant difference was detected in either beam balance (repeated measures two-way ANOVA: F_(1,20)_ = 0.04, p = 0.853) or ipsilateral foot faults (repeated measures two-way ANOVA: F_(1,20)_ = 0.08, p = 0.785) (data not shown). SAHA administration did reduce post-injury weight loss (interaction of group and day by repeated measures two-way ANOVA: F_(6,120)_ = 5.08, p<0.001) (Figure 5D), an effect opposite to that seen following VPA treatment. When these animals were tested in the Morris water maze, no significant influence of SAHA administration was observed in either learning (repeated measures two-way ANOVA: F_(1,20)_ = 1.66, p = 0.212; Figure 5E) or measures of memory during a 24 hr probe trial (latency to platform: Student's *t-test* P = 0.134; number of platform crossings: Mann Whitney Rank sum test P = 0.477).

### Valproate administered 30 min post-injury reduces cortical contusion volume

**Figure 6:**
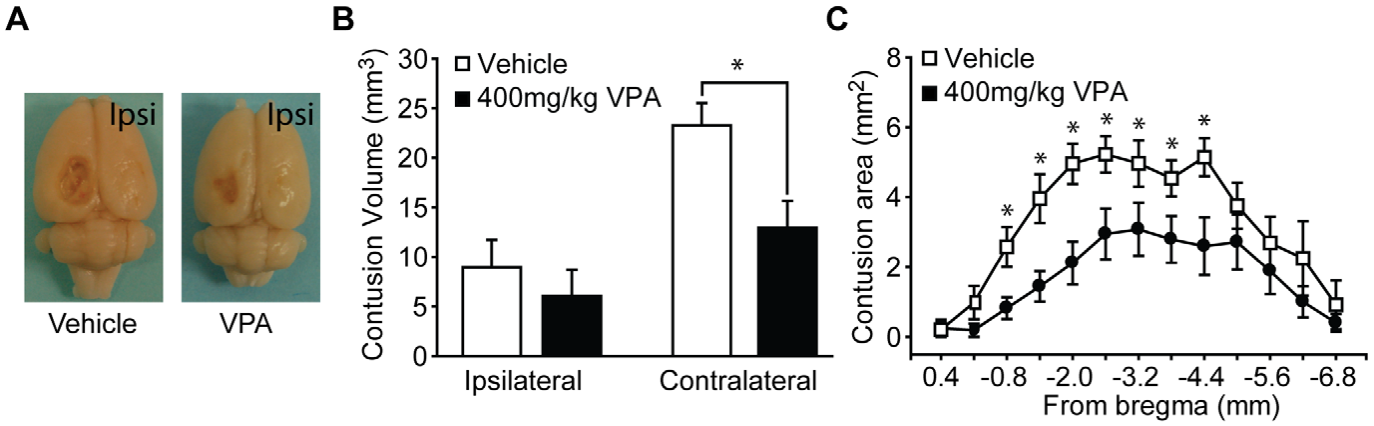
Post-injury administration of 400 mg/kg valproate reduces contusion volume. **A)** Representative photographs of the brains from a vehicle- and a 400 mg/kg valproate (VPA)-treated animal. Animals were killed 4 weeks post-injury. **B)** Quantification of the volume of lost cortical tissue revealed that 400 mg/kg VPA significantly reduced contralateral tissue loss. **C)** Rostral-caudal extent of the damage detected in the contralateral cortex from vehicle- and 400 mg/kg VPA-treated animals. Data are presented as the mean ± SEM. ≨︀, significant difference by repeated measures two-way ANOVA. *, P≤0.05.

Following the completion of the behavioral studies, VPA- and vehicle-injected animals were euthanized and brains removed for determination of contusion volume. Figure 6A shows photographs of the brains of a representative vehicle- and 400 mg/kg VPA-treated animal (30 min post-injury treatment initiation). The pictures show that the animal treated with 400 mg/kg VPA had contralateral cortical tissue loss that appears to be dramatically smaller in size than that seen in the vehicle-treated animal. Quantification of this tissue loss revealed that 400 mg/kg VPA significantly reduced contralateral tissue loss compared to animals receiving vehicle injections (Student's *t-test:* p = 0.014; Figure 6B). When examined over the rostral-caudal extent of the injury, it was found that 400 mg/kg VPA reduced the magnitude of damage primarily anterior to the contralateral injury core (two-way repeated measures ANOVA: F_(1,18)_ = 6.271, P = 0.021; Figure 6C). This protection on contralateral tissue loss was not observed in the animals treated with 100 mg/kg VPA (Student's *t-test:* p = 0.362), nor in the 400 mg/kg VPA-treated animals whose treatment was delayed by 3 hr following injury (Student's *t-test:* p = 0.963). Influences on ipsilateral tissue loss were not observed at any of the doses or times of administration tested.

### Improved spatial learning and memory are associated with preserved hippocampal MAP2 immunoreactivity

**Figure 7:**
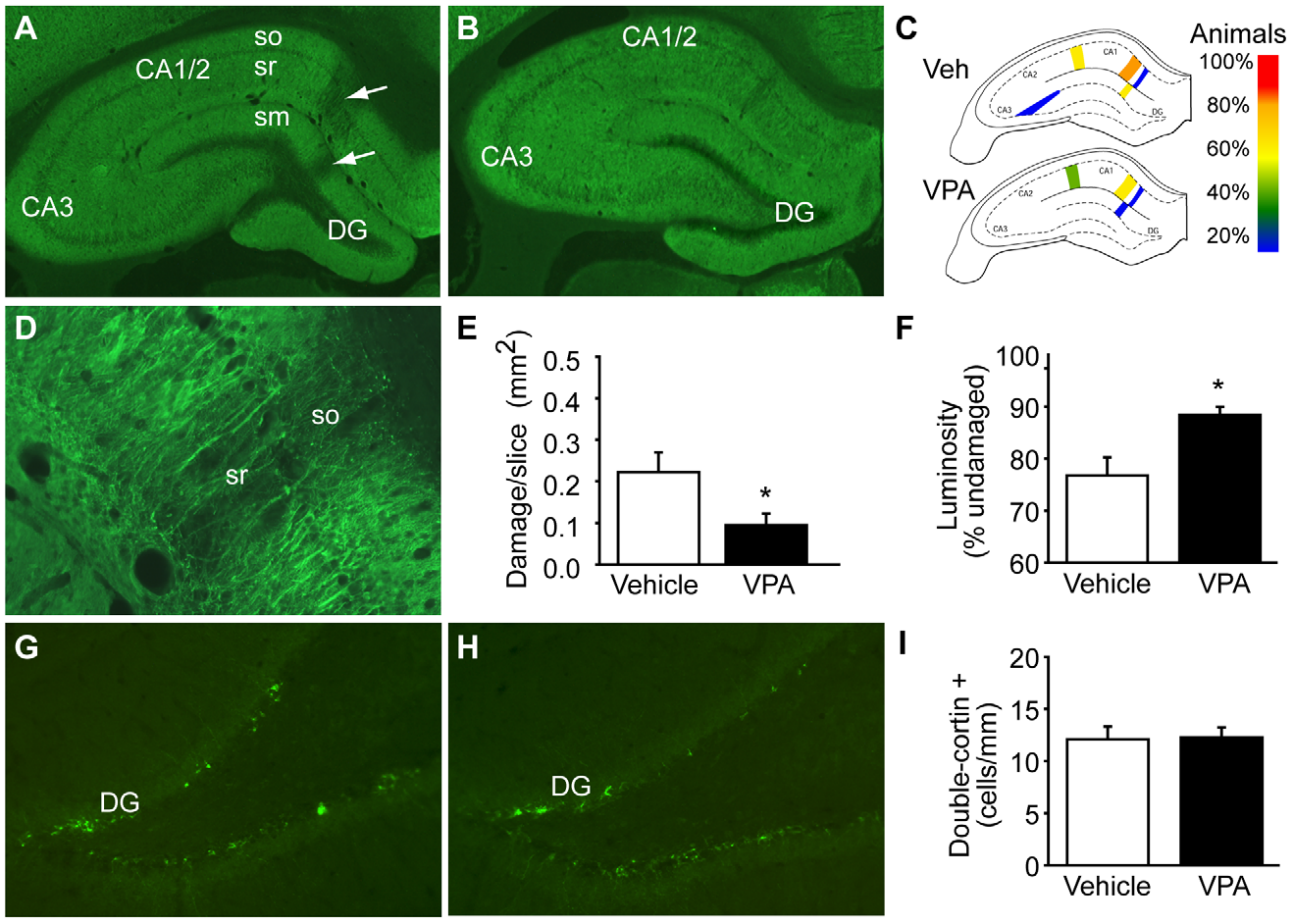
Post-injury treatment with 400 mg/kg valproate offers neuroprotection after TBI. Representative photomicrographs showing microtubule-associated protein 2 (MAP2, a dendritic marker) immunoreactivity in the ipsilateral hippocampi of injured animals treated with either **A)** vehicle or **B)** 400 mg/kg valproate (VPA). Arrows indicate areas of dendritic damage. **C)** Illustrations showing the location of, and percentage of animals with, reduced MAP-2 immunoreactivity. **D)** High magnification photomicrograph showing a representative area with disrupted MAP2 immunoreactivity. **E)** Summary data showing the average of the cumulative area of reduced MAP-2 immunoreactivity from the hippocampi of vehicle and 400 mg/kg VPA-injected injured animals. **F)** Average fluorescent intensity of the areas with reduced MAP-2 immunoreactivity from the hippocampi of vehicle- and 400 mg/kg VPA-treated animals. Fluorescent intensity of the damaged area was normalized within each section by comparison to the fluorescent intensity of an adjacent, undamaged area. Representative photomicrographs showing doublecortin immunoreactivity from a **G)** vehicle- and a **H)** 400 mg/kg VPA-treated animal. **I)** Summary data showing that treatment with 400 mg/kg VPA had no effect on the number of doublecortin positive cells by 4 weeks post-injury. Data are presented as mean ± SEM. *, P≤0.05. DG: dentate gyrus, so: stratum oriens, sr:stratum radiatum, sm: stratum molecular.

Valproate has been previously demonstrated to induce dendritic growth of cultured cells [42], [43] and TBI has been shown to cause disruption of hippocampal dendrites [20], [44]. As our Morris water maze results indicate that treatment of injured animals with 400 mg/kg VPA starting 30 min post-injury significantly improves hippocampal function, we questioned if this improvement was associated with preserved hippocampal dendritic integrity. To test this, immunohistochemistry was performed using the dendritic marker microtubule-associated protein 2 (MAP2). The tissue sections prepared following the completion of the behavioral studies were used for these experiments. Figure 7A shows a representative photomicrograph of a hippocampus from a vehicle-treated, injured animal immunoreacted for MAP2. Areas of reduced MAP2 immunoreactivity can be seen in both the stratum radiatum and stratum molecular. In contrast to that seen in vehicle-treated animals, these disruptions appeared to be reduced in rats treated 30 min post-injury with 400 mg/kg VPA (Figure 7B). When the location of disruptions were recorded, it was found that while most vehicle-treated animals had discernable damage adjacent to the CA1/CA2 (80% of all animals examined) and dentate gyrus (DG; 60% of all animals examined) subfields, these damages were observed less frequently in the 400 mg/kg VPA-treated animals (60% and 20%, respectively) (Figure 7C). In order to quantify the area and degree of MAP2 disruption, high magnification photomicrographs (representative image shown in Figure 7D) were taken, and the size and relative fluorescent intensity of the region of MAP2 loss measured as described previously [20]. Figure 7E shows that the cumulative area of TBI-associated hippocampal MAP2 disruption/section was significantly reduced in VPA-treated rats compared to that measured in vehicle-treated controls (Student's *t-test* p = 0.049). Furthermore, areas that had disruption were found to have increased MAP2 immunoreactivity (Student's *t-test* p = 0.016; Figure 7F), suggesting spared fibers.

In addition to influences on dendritic growth, chronic valproate treatment has been shown to enhance neurogenesis within the hippocampus [45] and has been demonstrated to reduce monocyte/macrophage accumulation [46]. To test if the rate of TBI-associated increased neurogenesis [47]–[49] was influenced by our VPA treatment regimen, the number of doublecortin positive cells was counted. Representative photomicrographs from a vehicle- (Figure 7G) and a VPA- (Figure 7H) treated animal are shown. No significant difference was detected between the two groups (quantified as # of cells/mm dentate gyrus: vehicle = 12.09 ±1.22; 400 mg/kg VPA = 12.28±0.95, P = 0.899; Figure 7I). Similarly, although a dramatic increase in CD68-immunopositive monocytes/macrophages was observed in the corpus callosum as a result of injury, this did not appear to be affected by VPA treatment (Figure S1).

## Discussion

Using a rodent model of TBI, we examined if post-injury VPA administration is neuroprotective, and improves motor and cognitive outcomes. Our preclinical findings show that post-injury systemic administration of VPA: 1) reduced cortical contusion volume, 2) improved blood-brain barrier integrity (BBB), 3) reduced hippocampal MAP2 disruptions, and 4) improved motor and cognitive function.

Valproate [2-propylpentanoic acid] (VPA) is a simple branched-chain fatty acid with well established efficacy for seizures [50]. It is also commonly prescribed for bipolar disorder, acute mania and migraines [10]. The therapeutic concentration of valproate is 40–100 µg/ml, and it has a serum half-life of 8–17 hours in adults. This therapeutic concentration is achieved by a loading dose (as low as 10 mg/kg) followed by maintenance doses (as high as 60 mg/kg). In rodents, an i.p. dose of 200 mg/kg gives rise to a peak serum concentration of 400 µg/ml 15 minutes post injection that rapidly decreases to about 40 µg/ml by 8 hr post-infusion [8]. In contrast, an injection of 400 mg/kg gives rise to a plasma concentration of approximately 150 µg/ml at 8 hr. A number of studies have utilized 300 mg/kg or 400 mg/kg and have observed HDAC and/or GSK-3 inhibition in the brain and other organs [8], [51]–[53]. Consistent with these studies, we found that 400 mg/kg VPA significantly increased histone acetylation, and reduced β-catenin phosphorylation in hippocampal tissue extracts (Figure 1).

Using a dose of 400 mg/kg, we observed that VPA improved motor function, and enhanced spatial learning and memory. Although the VPA-treated animals displayed a quadrant preference when assessed in a 24 hr probe trial, post-hoc analysis revealed that this difference was due to a preference for the target quadrant (I) relative to only the starting quadrant (III). This implies that although the VPA-treated, injured animals have better long-term memory for the platform position than do vehicle-treated injured animals, the performance of neither group was to the level typically observed for uninjured animals The improvement in motor and cognitive performance seen after acute VPA treatment suggests that this compound may reduce TBI-associated neuronal degeneration. Previous studies have reported that valproate can be neuroprotective, both for cells grown in culture and as well as in a rodent models of *status epilepticus* and ischemic stroke [42], [43], [52]. For example, Ren *et al* reported that treatment of rats with 300 mg/kg valproate following middle cerebral artery occlusion (MCAO) significantly reduced infarct size and improved neurologic recovery [52]. Consistent with this, our measurement of cortical contusion volume showed that injured animals treated 30 min following injury with 400 mg/kg VPA had significantly reduced cortical tissue loss than their vehicle-treated counterparts. In addition, we found that TBI-associated disruption of hippocampal MAP2 immunoreactivity, an indicator of dendritic damage, was also reduced in VPA-treated animals. This is consistent with previous reports that have indicated that treatment of neurons with valproate induces neurite outgrowth [42]. However, it is not known if the reduction in MAP2 disruption we observed represents a preservation of dendritic integrity, or is the result of dendritic outgrowth. In addition to influences on dendritic integrity, VPA treatment has been shown to enhance hippocampal neurogenesis [45]. Although we anticipated seeing an influence of VPA on the rate of hippocampal neurogenesis, our analysis of doublecortin positive cells examined after the completion of behavioral training did not reveal any significant increase in cell numbers relative to that observed in vehicle-treated, injured animals. It has been reported that doublecortin is expressed during the late mitotic and early post-mitotic stages of neurogenesis (1–3 weeks post proliferation) [54], [55]. As the animals used for this analysis were euthanized approximately 4 weeks after injury, it is possible that the neurons generated during the time of VPA administration (given during the first week of injury) had become synaptically integrated and were no longer doublecortin positive. Alternatively, as both cortical impact injury and behavioral training have also been shown to increase the rate of hippocampal neurogenesis [49], [56], this may have made it difficult to detect VPA-mediated increases.

At present, the mechanism(s) underlying the protective effects of VPA are not clear. As decreased histone acetylation has been observed following TBI [57] (Figure S2), VPA may offer protection, in part, by increasing histone acetylation and enhancing the expression of genes involved in neuronal plasticity and survival. Consistent with this, Shein et al., have shown that acute treatment of mice following TBI with the HDAC inhibitor ITF2357 reduces contusion volume and improves motor function [58]. Further, Lyeth and colleagues have shown that DMA-PB (a novel HDAC inhibitor) attenuates the TBI-associated decrease in histone acetylation and reduces microglia-mediated inflammation [59]. However, post-injury treatment with SAHA did not improve motor or cognitive function, suggesting that the HDAC-inhibiting activity of VPA is not sufficient to improve behavioral outcome following TBI. Consistent with the result obtained using SAHA, we have recently reported that post-injury treatment with sodium butyrate, a non-selective HDAC inhibitor, also failed to improve cognitive function when administered acutely following TBI [60]. In addition to inhibition of HDACs, the mood-stabilizing properties of valproate are thought to be due to its ability to inhibit GSK-3 [61]. Although the role of GSK-3 in TBI pathology has not been well described, this cascade has been shown to be critical for the regulation of ß-catenin-mediated gene expression and has been implicated in the mechanism of action of several neuropsychiatric drugs [62], [63]. Alam and colleagues have shown that valproate treatment enhances survival following lethal hemorrhagic shock with and without polytrauma (300 and 400 mg/kg, respectively), and that this protection is associated with nuclear translocation of ß-catenin and increased expression of bcl-2 [51], [64]. Recent studies have shown that valproate can also elicit neuronal growth via activation of ERK [42]. We have previously shown that the activation of ERK following TBI is neuroprotective, and its inhibition exacerbates TBI-associated motor and cognitive deficits [20]. Although our western blots did not reveal any significant increase in ERK phosphorylation in response to VPA injection, its activation at an earlier/later time point than that examined here (45 min post-injection) cannot be ruled out. Lastly, while not specifically examined in the current study, valproate has also been shown to enhance GABAergic neurotransmission. Previous studies have shown that administration of a GABA agonist acutely following injury can be used to reduce hyperactivity and improve neurological outcome [65]–[67]. It is therefore plausible that one or a combination of these mechanisms may underlie the beneficial influences of VPA we observed.

At the doses and dosing regimens employed, no adverse influences of VPA were observed except for an exacerbation of post-injury weight loss at the 400 mg/kg dose. TBI is known to cause an acute loss in body weight that is most likely due to reduced food consumption during the acute phase of injury [68]. Evaluation of body weight revealed that rats treated with 400 mg/kg VPA took approximately twice as long to return to their pre-injury weights. It has been reported that caloric restriction can be used to enhance neuronal plasticity, increase resistance to oxidative stress, to reduce insulin sensitivity and excitotoxicity [69], [70]. Consistent with these benefits, food restriction has been shown to be associated with a recovery of spatial memory following global ischemia [71]. Although we cannot rule out any contribution that the enhanced weight loss had on the cognitive improvements we observed, our time window studies suggest that this was unlikely to be a determining factor. This conclusion is based on our observation that while both 30 min and 3 hr post-injury groups had enhanced weight loss and motor skill improvement as a result of daily 400 mg/kg VPA injection, only the 30 min post-injury animals displayed improved spatial learning and memory.

TBI patients often have trauma to other organs, resulting in blood loss and hemorrhagic shock. Recently, Alam and colleagues have shown that acute valproate can be used to reduce the lethality of hemorrhagic shock in both rodent and swine models [51], [64]. These findings, along with our findings on the beneficial effects of VPA treatment on BBB function, cognitive improvement and neuronal protection, suggest that this drug may have benefit in the treatment of TBI and TBI with polytrauma. One potential limitation of valproate therapy to improve cognitive function is that it appears to only be effective when given less than 3 hr post-injury. Further experiments will be required to establish the exact time window, the best routes of administration, and if this improvement can be observed following different injury magnitudes [72].

## Supporting Information

Figure S1 Valproate does not appear to reduce infiltration of inflammatory cells. Valproate (VPA) has been demonstrated to reduce monocyte/macrophage accumulation following experimental autoimmune neuritis [46]. As increased monocyte infiltration has been observed following TBI, we investigated if 400 mg/kg VPA can reduce the number of these cells. Representative photomicrographs showing CD68 (a marker for circulating monocytes/macrophages) immunoreactivity in the ipsilateral hippocampi of sham, injured animals treated with vehicle, and injured animals treated with 400 mg/kg valproate (VPA). Dotted lines indicate the position of the hippocampal neuronal layers. Although dramatic infiltration of CD68-positive monocytes/macrophages can be observed in the corpus callosum (cc) following injury, no overt influence of VPA was observed. DG: dentate gyrus.
(7.29 MB TIF)

Figure S2 TBI decreases histone acetylation in the hippocampus. Jenkins and colleagues have recently demonstrated that controlled cortical impact injury causes a decrease in hippocampal histone acetylation in juvenile rats that is localized to the CA3 subfield [57]. To determine the influence of cortical impact injury on histone acetylation in adult rats, hippocampal tissue samples were prepared at various time points following injury and subjected to western blot analysis using antibodies that specifically detect either acetylated H3 or acetylated H4 histones. A) Representative western blot showing histone H3 and H4 acetylation in hippocampal protein extracts at different time points after TBI (n = 4/time point). Summary results of the changes in the acetylation for B) histone H3 (one-way ANOVA F(4,15) = 0.029, P = 0.998) and C) histone H4 (one-way ANOVA F(4,15) = 0.350, P = 0.840) after injury. In order to examine if histone acetylation changed in a localized manner, immunohistochemistry was performed on brain sections taken from sham-operated and 24 hr post-injury animals. Fresh frozen sections (20 µm in thickness) were prepared, mounted on gelatin-coated slides and fixed for 20 min in −20°C methanol. Anti-acetylated histone H3 antibodies (5 µg/ml in Tris-buffered saline containing 0.25% TX-100 and 5% normal goat serum) were incubated at room temperature for 3 hr followed by detection with an anti-rabbit antibody conjugated to Alexa488. Immunofluorescence was visualized using a UV microscope (Axiophot, Zeiss) and the appropriate filter sets. D) Representative images of acetylated H3 immunostaining within the hippocampi of a sham, and a 24 hr injured animal. E) High magnification images of the areas indicated in (D). Data are presented as mean ± SEM.
(9.30 MB TIF)
