# Supplementary figures and images for: Valproate Administered after Traumatic Brain Injury Provides Neuroprotection and Improves Cognitive Function in Rats
Source: PLoS One. 2010 Jun 30;5(6):e11383. doi: 10.1371/journal.pone.0011383 (PMC2894851; doi:10.1371/journal.pone.0011383)

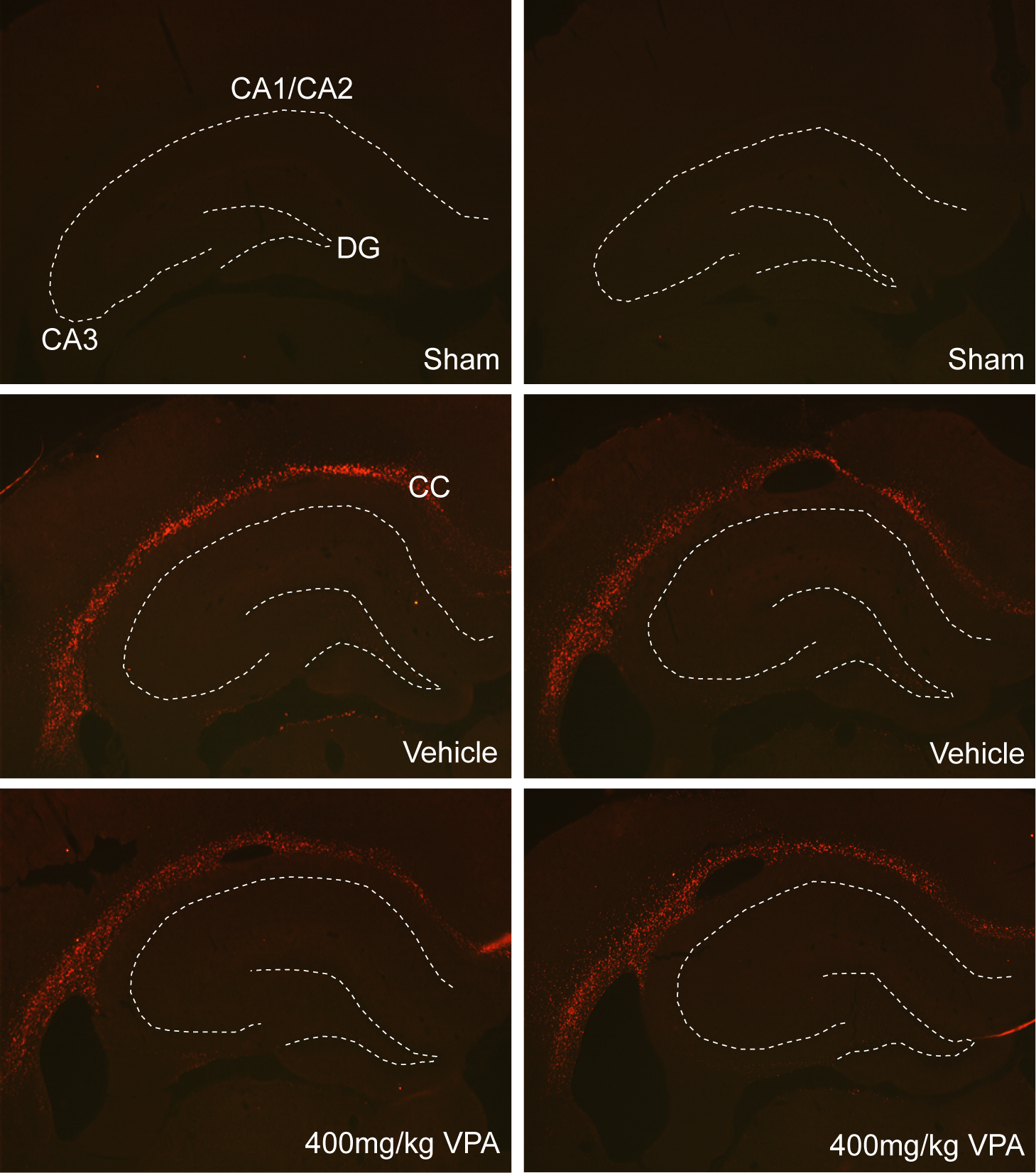

Supplement: Figure S1 — Valproate does not appear to reduce infiltration of inflammatory cells. Valproate (VPA) has been demonstrated to reduce monocyte/macrophage accumulation following experimental autoimmune neuritis [46]. As increased monocyte infiltration has been observed following TBI, we investigated if 400 mg/kg VPA can reduce the number of these cells. Representative photomicrographs showing CD68 (a marker for circulating monocytes/macrophages) immunoreactivity in the ipsilateral hippocampi of sham, injured animals treated with vehicle, and injured animals treated with 400 mg/kg valproate (VPA). Dotted lines indicate the position of the hippocampal neuronal layers. Although dramatic infiltration of CD68-positive monocytes/macrophages can be observed in the corpus callosum (cc) following injury, no overt influence of VPA was observed. DG: dentate gyrus. (7.29 MB TIF) [file pone.0011383.s001.tif]

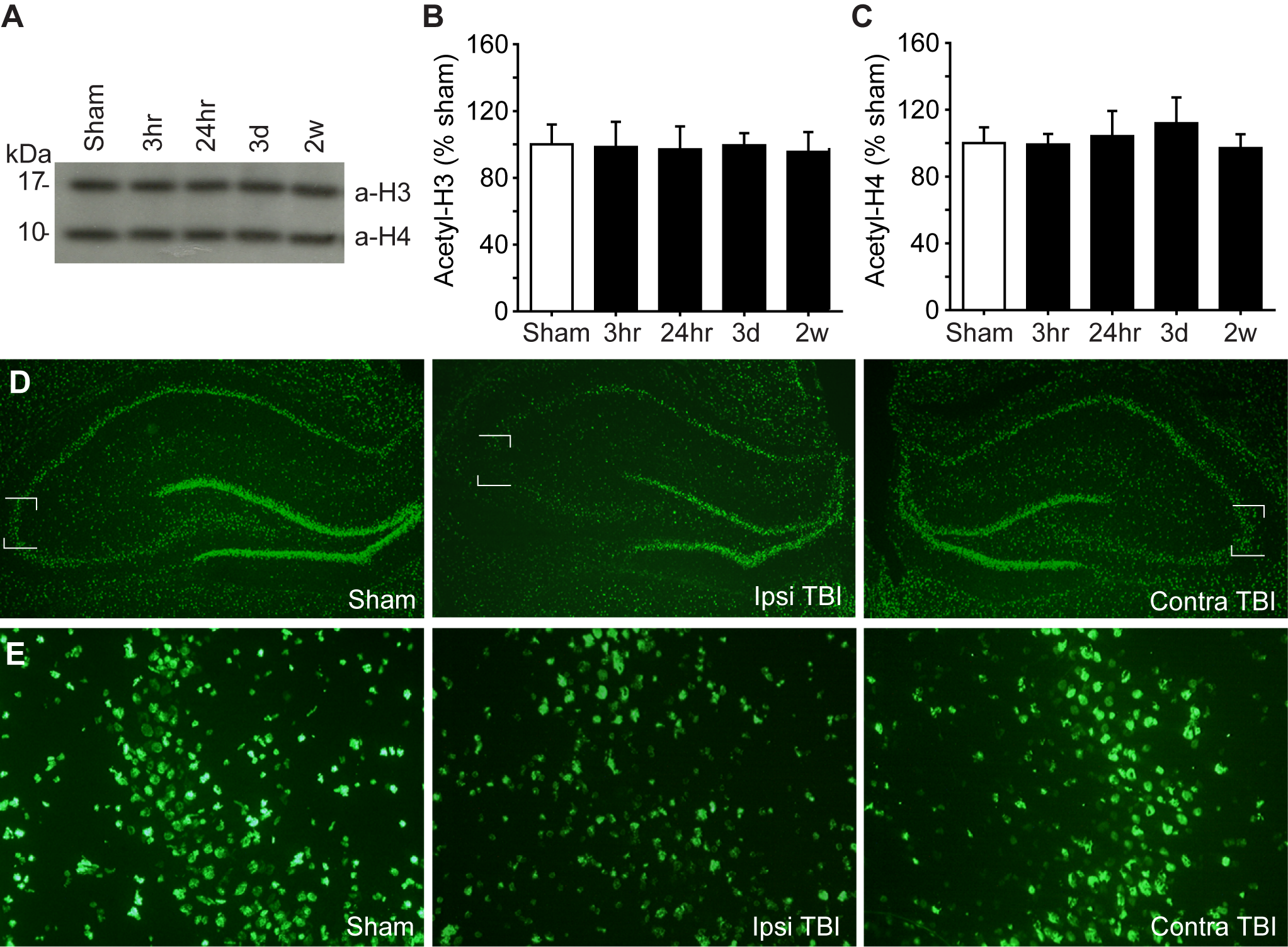

Supplement: Figure S2 — TBI decreases histone acetylation in the hippocampus. Jenkins and colleagues have recently demonstrated that controlled cortical impact injury causes a decrease in hippocampal histone acetylation in juvenile rats that is localized to the CA3 subfield [57]. To determine the influence of cortical impact injury on histone acetylation in adult rats, hippocampal tissue samples were prepared at various time points following injury and subjected to western blot analysis using antibodies that specifically detect either acetylated H3 or acetylated H4 histones. A) Representative western blot showing histone H3 and H4 acetylation in hippocampal protein extracts at different time points after TBI (n = 4/time point). Summary results of the changes in the acetylation for B) histone H3 (one-way ANOVA F(4,15) = 0.029, P = 0.998) and C) histone H4 (one-way ANOVA F(4,15) = 0.350, P = 0.840) after injury. In order to examine if histone acetylation changed in a localized manner, immunohistochemistry was performed on brain sections taken from sham-operated and 24 hr post-injury animals. Fresh frozen sections (20 µm in thickness) were prepared, mounted on gelatin-coated slides and fixed for 20 min in −20°C methanol. Anti-acetylated histone H3 antibodies (5 µg/ml in Tris-buffered saline containing 0.25% TX-100 and 5% normal goat serum) were incubated at room temperature for 3 hr followed by detection with an anti-rabbit antibody conjugated to Alexa488. Immunofluorescence was visualized using a UV microscope (Axiophot, Zeiss) and the appropriate filter sets. D) Representative images of acetylated H3 immunostaining within the hippocampi of a sham, and a 24 hr injured animal. E) High magnification images of the areas indicated in (D). Data are presented as mean ± SEM. (9.30 MB TIF) [file pone.0011383.s002.tif]
